# Morphostructural Damage in Food-Spoiling Bacteria due to the Lemon Grass Oil and Its Vapour: SEM, TEM, and AFM Investigations

**DOI:** 10.1155/2012/692625

**Published:** 2012-10-02

**Authors:** Amit Kumar Tyagi, Anushree Malik

**Affiliations:** Applied Microbiology Laboratory, Centre for Rural Development and Technology, Indian Institute of Technology Delhi, New Delhi-110 016, India

## Abstract

In this study, antimicrobial activity and morphostructural damages due to lemon grass oil (LGO) and its vapour (LGOV) against *Escherichia coli* strains were investigated. Minimum inhibitory concentration (MIC) and minimum bactericidal concentration (MBC) of LGO were determined by broth-dilution method to be 0.288 mg/mL and 0.567 mg/mL, respectively. Furthermore, the zone of inhibition (45 mm) due to the vapour phase antimicrobial efficacy evaluated using disc volatilization assay was compared with that using disc diffusion assay (i.e., 13.5 mm for the same dose of oil). The morphological and ultrastructural alterations in LGO- and LGOV-treated *E. coli* cells were studied using scanning electron microscopy (SEM), transmission electron microscopy (TEM), and atomic-force microscopy (AFM). In SEM observation, LGO-treated cells appeared to be aggregated and partially deformed, while LGOV-treated cells lost their turgidity, and the cytoplasmic material completely leaked from the cells. In TEM observation, extensive intracytoplasmic changes and various abnormalities were observed in LGOV-treated cells more than LGO-treated cells. Significant variations in the height and root mean square values of untreated, LGO-, and LGOV-treated *E. coli* cells were noticed by AFM. Present results indicate that LGO is highly effective against *E. coli* in vapour phase.

## 1. Introduction

Microbial contamination of food is increasingly becoming a cause of concern for human health [1]. Recent findings indicate that the food-producing animals, improper cultivation, and unhygienic handling at critical stages of food production can represent some of the most important sources for the entry of virulent and sometimes multidrug-resistant E. coli strains in the food chain [2, 3]. Therefore, there is a continuous need to develop novel antimicrobial agents to minimize the food contamination as well as the threat of further antimicrobial resistance [4]. In this regard, natural antimicrobials such as essential oils have attracted considerable attention due to the increased consumer awareness on the aspects of food quality and safety [5–7].

Although the essential oils have high efficiency against the foodborne pathogen and spoilage microorganisms in liquid phase, this effect in food is only achieved with higher concentrations of essential oils as compared to the MIC in nutrient media [8, 9]. Hence, in spite of ample research on the use of essential oils, the application in food preservation is yet to be developed due to the adverse impact of higher concentrations on the organoleptic properties [10]. For reducing the sensory effect, one of the alternative approaches may be the use of essential oil in the vapour phase [11]. Essential oil in vapour phase could be highly effective against foodborne pathogens and spoilage bacteria at relatively lower concentrations [12] than the liquid phase, thereby causing minimum effect on organoleptic properties. This would also be amenable to the upcoming MAP or modified atmospheric packaging [13] and nanoencapsulation technology [14].

Lemon grass oil (LGO) is a natural plant extract, whose antimicrobial properties against *Candida albicans* in liquid as well as in vapour phase have been studied by our group [15]. Nevertheless, to establish the antibacterial efficacy as well, detailed knowledge of the effects of LGO and LGOV on bacterial cell structures is very important. However, so far the studies relating to the cellular morphology of bacteria and the subsequent disruption of their integrity as a result of certain chemicals have been performed using scanning electron microscopes and transmission electron microscopes. Atomic force microscopy (AFM) is a powerful tool in microbiology to study the structure and properties of microbial surfaces at the nanometer level under physiological conditions [16]. Since its invention in 1986, AFM has widely been applied in a variety of nanometer scale investigations in biosciences, including imaging of bacteria [17–19]. The advantages of this technique are that the height and size of the observed objects can be measured precisely. Furthermore, the use of AFM allows the direct observation of processes occurring on the surface of living bacteria treated with the antimicrobial compounds [20–22]. Assessment of damage to bacteria by control agents including the morphological changes to *Escherichia coli* caused by the antibiotic cefodizime [23], and the *Staphylococcus aureus* response to vancomycin [24], were done by studying antibacterial peptides using AFM [25]. Nevertheless, the use of such powerful techniques in assessing the cell damage induced by essential oils is rarely seen. As of yet, AFM has not been used to examine the mechanism of action of LGO against *E. coli*. 

The aim of the present study is to perform a high resolution investigation on the surface and morphological alterations induced in *E. coli *cells after exposure to LGO and LGOV. It is envisaged that by employing a battery of imaging techniques like scanning electron microscopy (SEM) and transmission electron microscopy (TEM) along with AFM, interaction of LGO and LGOV with bacterial cell surfaces could be elucidated. 

## 2. Material and Methods

### 2.1. Materials and Bacterial Culture Preparation

LGO was procured from Natural Aromatics Pvt. Td., New Delhi (India) where the steam-distillation method has been used for LGO preparation from lemon grass (*Cympobogon citratus*). Growth media, DMSO, and Tween 80 were purchased from Himedia and Qualigens, India, respectively, while ethanol and diethyl ether were purchased from Merck, India. 

*E. coli *DH5*α* and *E. coli *ATCC 25922 strains collected from the central microbial culture facility, Department of Biotechnology and Biochemical Engineering, Indian Institute of Technology Delhi, New Delhi India, and Himedia Pvt. Ltd. (India), respectively, were grown in Mueller-Hinton broth (MHB) medium at 30°C 24 h in an orbital shaking incubator (Scigenics India Pvt. Ltd., India) at 180 rpm. Cells were harvested by centrifugation, suspended in sterile distilled water, and used immediately.

### 2.2. Antimicrobial Assays

#### 2.2.1. Determination of MIC and MBC by Broth Dilution Method

Minimum inhibitory concentration (MIC) and minimum bactericidal concentration (MBC) of LGO were determined by broth dilution assay [26]. A range of essential oil concentrations (0.27–18 mg/mL) was prepared in 100 mL Mueller-Hinton broth (MHB) medium. To enhance oil solubility, Tween 80 was included at a final concentration of 0.05% (v/v). Each flask was inoculated with 10^6^ cfu/mL of each *E. coli *strain. Flasks containing only Tween 80 (without essential oil) and MHB were used as control. The flasks were incubated at 30°C in an orbital shaking incubator (180 rpm) for 24 h. One mL of culture was taken from each flask for serial dilution to make the inoculum of 10^6^ cfu/mL and inoculated on MHA plates at 30°C for 24 h. The plates were observed and MIC was determined. Furthermore the flasks having lesser essential oil concentration than MIC level were subcultured four times and inoculated on MHA plate for MBC determination.

#### 2.2.2. Disc Diffusion Method

Disc diffusion method was employed for the determination of antimicrobial activities of the LGO in liquid phase [27]. Briefly, a 100 *μ*L portion of each suspension containing approximately 10^6^ cfu/mL was spread over the surface of the MHA plate and allowed to dry. A paper disc (diameter 6 mm, Sigma-Aldrich, India) was impregnated with 10 *μ*L essential oil on each disc and placed on the inoculated plates. These plates, after staying at 4°C for 2 h, were incubated at 37°C for 24 h for bacterial growth. The diameters of the inhibition zones were measured in millimetres. Volume of essential oils tested was varied (20, 40 *μ*L) by using an appropriate number of sterile discs. 

#### 2.2.3. Disc Volatilization Method

Standard experimental set-up as described by [28] was used. Briefly, a 100 *μ*L portion of each suspension containing approximately 10^6^ cfu/mL was spread over the surface of the MHA plate and allowed to dry. A paper disc (diameter 6 mm, Sigma-Aldrich, India) was laid on the inside surface of the upper lid and 10 *μ*L essential oil was placed on each disc. The plates inoculated with microorganisms were immediately inverted on top of the lid and sealed with parafilm to prevent leakage of essential oil vapour. Plates were incubated at 30°C for 24 h and the diameter of the resulting inhibition zone in the bacterial lawn was measured. Volume of essential oils tested was varied (20, 40 *μ*L) by using an appropriate number of sterile discs. 

### 2.3. Preparation of **E. coli ** Sample for Morphological Study

The *E. coli* ATCC 25922 cells were incubated for 14 h in MHB at 30°C and 180 rpm. The suspension was divided into two portions. In one portion, LGO at MIC level (0.288 mg/mL) was added and another portion was left untreated as a control. The resuspension was incubated at 30°C for 4 h. 

For investigating the effect of LGO vapour, 1 mL of *E. coli* cell suspension (10^6^ cells/mL) was inoculated on an MHA plate and incubated at 30°C for 12 h. These Pregrown cells were treated with LGOV (0.30 mg/mL) for 4 h. The treated cells were then collected gently with the help of a brush from the petri plate and collected in a separate test tube. All the treated cells were harvested by centrifugation and were prefixed with a 2.5% glutaraldehyde solution overnight at 4°C. After this, the cells were again harvested by centrifugation and washed three times with 0.1 M sodium phosphate buffer solution (pH 7.2). Now each resuspension was serially dehydrated with 25%, 50%, 75%, 90%, and 100% ethanol, respectively. Then, cells were dried at “critical point.” 

For SEM, a thin film of cells was smeared on a silver stub. The samples were gold covered by cathodic spraying (Polaron gold). Finally, the morphology of the *E. coli *cells was observed on a scanning electronic microscope (ZEISS EVO 50). The SEM observation was done under the following analytical condition: EHT = 20.00 kv; WD = 10 mm; signal *A* = SE_1_. 

For TEM, the pellet was post-fixed in 1% osmium tetraoxide for 30 min, washed with phosphate buffer solution (pH 7.2), serially dehydrated, in ethanol and embedded in Epon-Araldite resin for making the blocks of the cells pellet. Ultra-thin (50–100 nm) sections of the cells were stained with uranyl acetate and lead citrate and observed under a Philips transmission electron microscope (CM-10) at 100 ev and direct magnification of 50.00 k.

The AFM images were taken employing the Veeco Metrology Group nanoscope IIIa operating in contact mode. In this mode of operation, a silicon nitrite tip with a force constant of 0.58 N/m was used. For AFM mounting of *E. coli* cells, glass substrates were employed. Ten micro-litres of each LGO-treated, LGOV-treated and untreated *E. coli* cell suspension was mounted on a glass substrate. After air-drying, the cells were imaged in air with AFM in tapping mode.

### 2.4. Statistical Analyses

All the experiments were done in triplicate and the data presented here represents the mean of three replicates. Data related to the zone of inhibition were subjected to analysis of variance (one-way ANOVA) in Duncan's multiple range test using SPSS (version 10) statistical software. The differences with *P* < 0.05 were considered significant. 

## 3. Results and Discussion 

### 3.1. Antimicrobial Assays

#### 3.1.1. Determination of MIC and MBC of LGO against **E. coli**

MIC of the LGO was determined against *E. coli*. The oil exhibited concentration-dependent inhibition of growth. A 0.288 mg/mL concentration of LGO was enough for complete growth inhibition of both *E. coli *strains. Minimum bactericidal concentration (MBC) is defined as the lowest concentration of oil resulting in the death of 99.9% of the inoculum (Burt, 2004). MBC of LGO for both *E. coli *strains was 0.567 mg/mL. In our previous studies, we observed that LGO had strongest antimicrobial activity against *C. albicans *and *P. fluorescens* as compared to mentha (*Mentha piperita*) and eucalyptus (*Eucalyptus globulus*) essential oils. The MIC of LGO against *Candida albicans *[15] was also similar (288 mg/L) to the present study while that against *Pseudomonas fluorescens* [29] was higher (567 mg/L). The MIC of *E. globulus* oil for *E. coli *DH5*α* and *E. coli *ATCC 25922 was several times higher (4.5 mg/L) than LGO [11].

Table 1 shows the MIC of *Cymbopogon* sp. oil and its constituents as reported by other authors [30–34] for different strains of *E. coli*. As is evident from this table, the MIC of different species of *Cymbopogon* sp. was found to vary from 0.12% to 0.8%. Hence, the MIC for LGO obtained in the present study (0.032%) is substantially lower than that reported earlier. Also, the MIC of LGO constituents such as Limonene, Linalool, *α*-pinene, and *β*-pinene against the same strain of *E. coli* used in the present study (ATCC 25922) was reported to vary from 1.25 mg/mL to 20 mg/mL in the previous study [32]. Nevertheless, the MIC of one of the major and active component of LGO, that is, geraniol, has been reported to be quite low (20 *μ*g/mL) for certain *E. coli* strains [33]. This is in agreement with the previous reports stating that acyclic *α*, *β*-unsaturated monoterpene aldehydes geranial and neral possess the most significant antimicrobial activity among the LGO constituents. The above discussion indicates that there is a great potential for LGO to be utilized as an antmicrobial or food preservative agent. 

#### 3.1.2. Zone of Inhibition due to the LGO and LGOV

**Figure 1:**
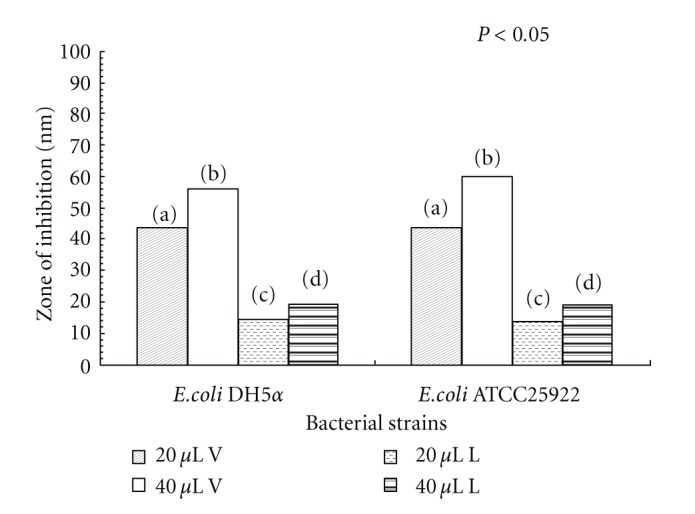
Zone of inhibition due to lemon grass oil (L) and lemon grass oil vapours (V) at different concentrations (20 *μ*L and 40 *μ*L) The bar of treatment followed by the same letter did not differ significantly by Duncan's multiple range test (DMRT, *P* < 0.05); LSD, least significant difference by ANOVA.

Comparison of the antibacterial activity of LGO (through Disc diffusion method) and LGOV (through Disc volatilization method) yielded interesting results. When* E. coli* cells were exposed to the same concentration of LGO and LGOV (20 *μ*L), the resulting zone of inhibition was found to be 13.5 mm and 44 mm, respectively. Similarly, at 40 *μ*L concentration also the inhibition zone due to vapours was significantly higher than that produced by the oil (Figure 1). These results show that LGOV exerted significantly higher antibacterial activity as compared to the LGO in liquid phase at the same concentration. 

Inouye et al. [35] studied the vapour phase antimicrobial activity of 72 essential oils. They found that some oils showed weak contact activity by the agar diffusion assay but strong vapour activity by the box vapour assay. However, they observed no difference in activity between the agar diffusion assay and box vapor assay for LGO. On the other hand, Suhr and Nielsen [36] reported that LGO-containing citral was most effective when added as volatiles as compared to direct contact assay. Comparing the present results with our previous reports, it is seen that the same concentration of the LGOV (20 *μ*L) produced 44 mm zone of inhibition in *E. coli* strains while producing 80 mm in *C. albicans* [15] and 26 mm in *P. fluorescens* [29]. Nevertheless, it is evident that in all the cases, LGO produced smaller inhibition zones as compared to LGOV, thus establishing the superior performance of the latter. This can be attributed to variation in chemical composition as well as mode of action of LGO and LGOV. Based on our previous investigations [15, 37] wherein the chemical composition of LGO and LGOV was analyzed by GC-MS and SPME-GCMS, respectively, it can be stated that both of them contained the oxygenated monoterpenes dominated by citral. Nevertheless, in the vapour phase certain other constituents such as limonene were also enriched. It is possible that these monoterpenes had better diffusibility in the gaseous phase as compared to the liquid oil phase [38]. To further differentiate the effect of LGO and LGOV on cell morphology and ultrastructure, SEM, TEM, and AFM observations were done.

### 3.2. Morphological Alteration of **E. coli**

#### 3.2.1. Scanning Electron Microscope (SEM) Observation

**Figure 2:**
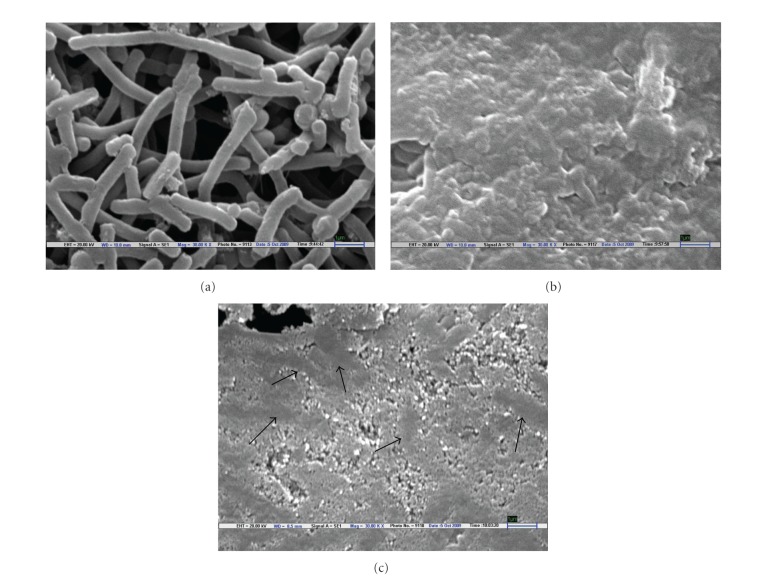
Scanning electron micrographs of untreated and treated *E. coli* cells. (a) Untreated cells with normal smooth surfaces (×30.00 K). (b) Shrunken, aggregated, and partially deformed LGO-treated cells (×30.00 K). (c) Completely destroyed and ruptured LGOV-treated cells (×30.00 K).

Bacterial cells treated with LGO at MIC level underwent considerable morphological alterations in comparison to the control when observed by a scanning electron microscope (Figure 2). Control *E. coli* cells appeared intact, rod shaped, separated from each other, turgid, and complete with smooth surface (Figure 2(a)) while the LGO- (0.288 mg/mL) treated cells appeared to be aggregated and partially deformed (Figure 2(b)). It seems that the cytoplasmic material of the bacterial cells had leaked and the aggregate cells appeared as sludge (Figure 2(b)). Similar observations indicating the aggregation of bacterial cells as a stress response upon exposure to antimicrobial compounds have been reported earlier [39]. Devi et al. [40] observed the effect of eugenol (clove essential oil) on *Salmonella typhi* cell surface by SEM, where the eugenol-treated bacterial cells showed deformation in their surface. The authors also visualized disruption of the bacterial membrane and a complete loss of membrane integrity by treatment with 1% (v/v) eugenol. Park et al. [41] noticed that citral, which is one of the major constituent of LGO [15] induced shrinkage and partial distortion of *Trichophyton mentagrophytes* hyphae at a very low concentration (0.09 mg/mL). 

When* E. coli* was exposed to LGOV, the cells were completely destroyed (Figure 2(c)). The SEM pictures clearly reveal that cells lost the turgidity and the cytoplasmic material completely leaked from the cells. In fact, only the ghost cells were left (shown by arrows) with apparent cellular debris (Figure 2(c)). Such extensive destruction of the cells is rarely demonstrated. Braga and Ricci [42] noticed such flattening and emptying in *E. coli* cells at Supra-MIC levels of the antimicrobial agent cefodizime. Da-Silva and Teschke, [43] showed that cells were lysed and only the “footprints” of lysed bacteria could be observed through AFM after 30 min incubation with antimicrobial peptide (PGLa). Sondi and Salopek-Sondi [44] observed that *E. coli* cells treated with silver nano-particles showed the formation of “pits” in their cell walls. LGOV evoked extensive cell damage similar to that observed with other established antimicrobial agents. Furthermore, the SEM micrographs confirmed the higher efficacy of LGOV as compared to LGO in liquid phase.

#### 3.2.2. Transmission Electron Microscope (TEM) Observation

Further evidence of antibacterial potential of LGO and LGOV has been obtained by TEM study. Untreated cells were studied as a control to ensure that the observed differences between control and the treated bacterial cells were indeed due to the effect of LGO/LGOV and not to the preparation method. 

**Figure 3:**
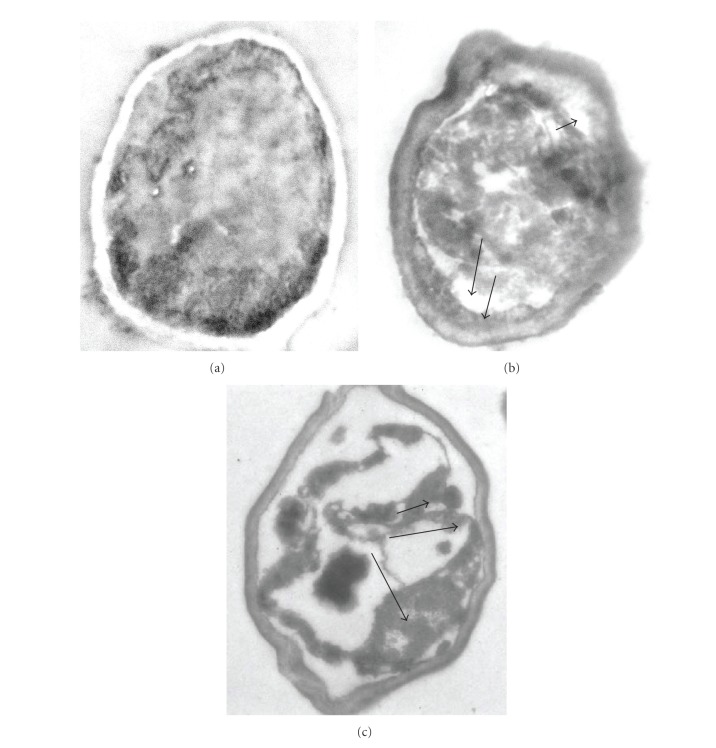
Transmission electron micrographs of untreated and treated *E. coli* cells. (a) Untreated *E. coli* cells having a regular outlined cell wall, plasma lemma lying closely to the cell wall, and regularly distributed cytoplasm. (b) LGO-treated *E. coli* cells having variable cell wall thickness appearing disrupted and variable periplasmic spaces (shown by arrows). (c) LGOV-treated cells having extensive internal damage, unsymmetrically distributed cytoplasm, and larger and irregular periplasmic spaces (shown by arrows).

TEM photomicrographs of untreated *E. coli* cells shows a regular outlined cell wall, plasmalemma lying closely to the cell wall, and some dense bodies regularly distributed over the cytoplasm (Figure 3(a)). Electron microscopy revealed that some of the oil-treated cells still retained a cell wall structure similar to untreated cells; however, in the majority of the cells, cell wall thickness varied and occasionally it appeared disrupted (Figure 3(b)). Extensive internal damage and a wide range of abnormalities were observed in the vapour-treated cells (Figure 3(c)). As shown in Figure 3, plasmalemma was damaged and became irregular in the treated cells (Figure 3(c)). Periplasmic space was altered and it became larger and irregular. Intracytoplasmic changes were noticed and the cytoplasm appeared very dense at certain locations and hence unsymmetrically distributed in the cell (Figure 3(c)). Mostly, coagulated material accumulated close to the cell wall and near the apical ends (Figure 3(c)). At certain locations, the cell envelope was damaged (Figure 3(c)) and the leakage of intracellular contents and emptying of the cells was evident. This can also result from alteration in membrane permeability leading to draining out of the inner contents while the main structure of the outer membrane still remains intact. Such observations were earlier recorded by Yi et al. [45] that tea-Polyphenols- (TP, 0.75 mg/mL) treated *Pseudomonas aeruginosa* showed alteration in the integrity of the outer membrane (OM) and disruption of cell walls. Both the OM an inner membrane (IM) permeation was demonstrated by increased fluorescence of the NPN (1-N-phenyl-naphthylamine) probe and the 4-methylumbelliferone, respectively, implying NPN uptake into cells through the OM and *β*-galactosidase release through the IM. This means that the TP damaged the OM and IM. However, optical density values at 260 nm generated little change in TP-treated samples, indicating that no large molecules, such as DNA and RNA, were released and the main structure of the bacterial membrane was still intact. The electron micrographs of chitosan-treated *E. coli* cells showed disrupted OM covered by an additional tooth-like layer indicating that the mechanism of the antibacterial activity of chitosan is through membrane damage [46]. Castillo et al. [47] also demonstrated that the antimicrobial action of arginine-based surfactant C3 (CA) 2 preferentially against Gram-negative bacteria (*E. coli*) is mediated through strong initial binding to the surface lipopolysaccharides and subsequent partitioning into the cell membrane to cause membrane damage, followed by cell death. Bactericidal activity of lipophilic monoterpenes (which are a major constituent of essential oils) is related to their capability to deeply interact with and affect the molecular structure of lipidic bilayers. Cristani et al. [48] have shown that *p*-cymene and carvacrol are more active against the Gram-negative *E. coli* as they markedly affect membrane lipid composition, taking the place of lipid molecules, and are strongly absorbed by lipidic membranes. On the other hand, antimicrobial activity of terpenes such as thymol, which possesses discrete lipophilic characteristics and a detectable water solubility, may be potentiated by the fact that they can migrate across the aqueous extracellular medium, interact with, and damage lipidic membranes. Since the outer layer of the Gram-negative outer membrane is composed primarily of lipopolysaccharide molecules and forms a hydrophilic permeability barrier providing protection against the effects of highly hydrophobic compounds [49], *E. coli* may exhibit low sensitivity to the cytotoxic effect of the highly lipophilic monoterpenes. Nevertheless, in the present study *E. coli* was found to be highly susceptible to low concentrations of LGOV as well as LGO. This could be attributed to the presence of significant amounts of citral both in LGO oil as well as the vapour [15, 37]. Strong antimicrobial properties of citral are already well documented. Among the four terpenes (citral, eugenol, *α*-terpineol, and nerolidol) tested in the antifungal assay against *Trichophyton mentagrophytes*, Park et al. [41] noticed the lowest MIC for citral. Furthermore, the authors also noticed extensive and irreversible cell membrane and organelles damage by exposure to 0.2 mg/mL citral through SEM and TEM observations while for other terpenes such damage was observed at much higher concentrations. The higher potency of the aldehyde (citral) is related to its higher lipophilicity enhancing its interaction and thereby inducing higher membrane damage. 

Based on the TEM observations and above discussion, it can be concluded that whilst the cell envelop was damaged at certain locations in the-LGOV-treated cells, the overall boundary of the damaged cells was retained. This indicates that the cell outline was probably maintained but the cells collapsed and turgidity was lost due to leakage of the cytoplasm. This correlates well with the ghost cells or footprints (boundaries intact but height flattened) of the cells observed in the SEM of the vapour-treated cells. To confirm this hypothesis, more detailed investigations are needed that can reflect upon the third dimension, that is, the height and roughness of the cells.

#### 3.2.3. Atomic Force Microscope (AFM) Observation

**Figure 4:**
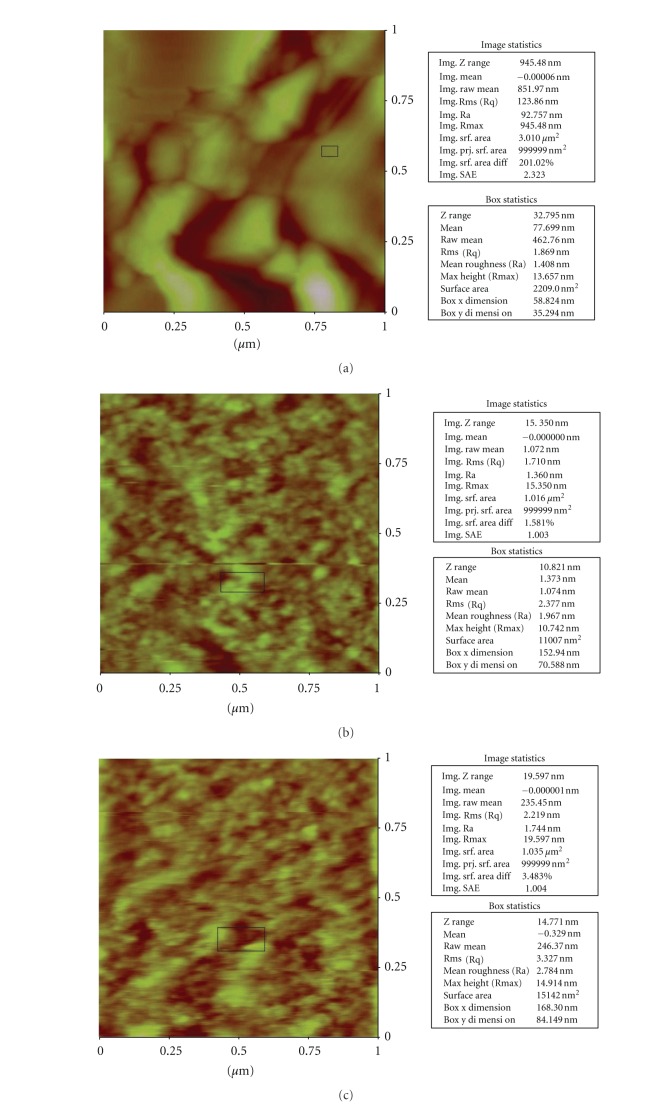

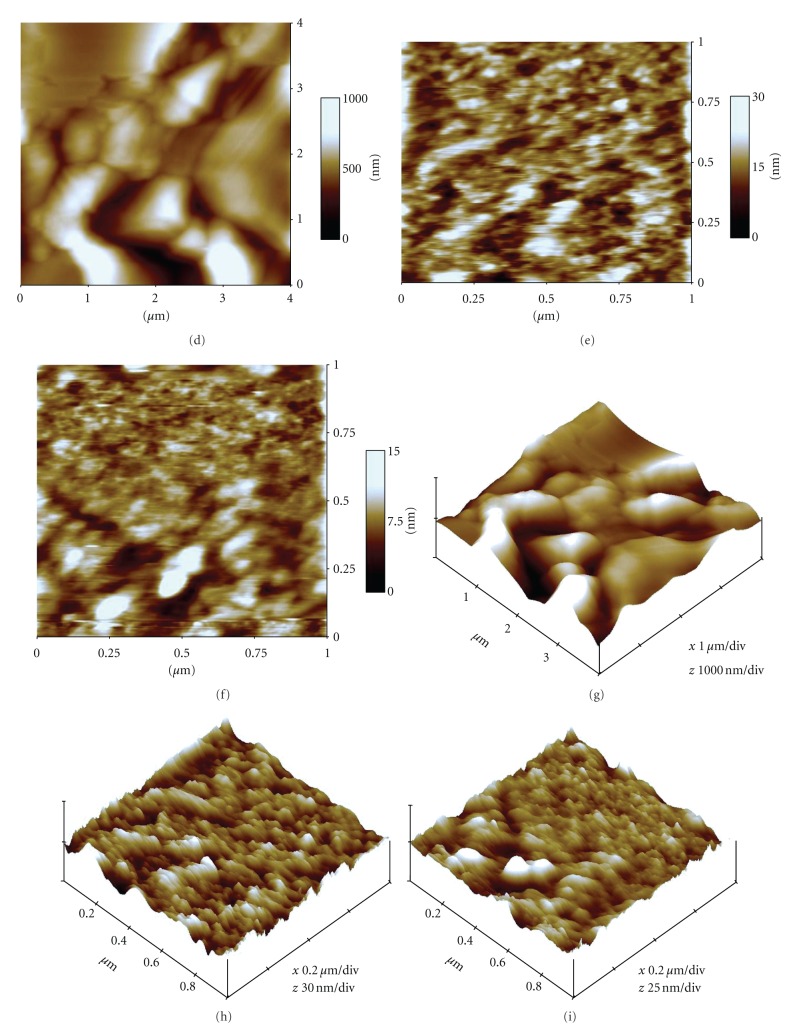
Atomic force micrographs showing variation in image statistics, height, and three-dimensional view of untreated, LGO-treated and LGOV-treated *E. coli* cells. (a) The root mean square values and surface area difference of untreated *E. coli* cells (rms 1.86 nm, sad 201.02%). (b) LGO-treated (rms 2.33 nm, sad 1.58%). (c) LGOV-treated (rms 3.32 nm, sad 3.48%). (d) Height of the untreated *E. coli* cells from the glass surface (h 450 nm). (e) LGO-treated (h 14 nm). (f) LGOV-treated (h 7 nm). (g) *Z*-axis value for three-dimensional structure of untreated *E. coli* cells (*z* 1000 nm/div). (h) LGO-treated (*z* 30 nm/div). (i) LGOV-treated (*z* 25 nm/div).

AFM of treated and untreated *E. coli* cells also show the evidences of change in morphology due to the antibacterial activity of the LGO and LGOV that corroborates with the SEM and TEM results. Significant variations in the height of the *E. coli *cells from the glass surface have been recorded. The height of untreated, LGO-treated and LGOV-treated was found to be 450 nm (Figure 4(d)), 14 nm (Figure 4(e)), and 7 nm (Figure 4(f)), respectively. The shape of the treated cells could be justified with this height measurement. In accordance with the SEM results, the height of LGO-treated shrunken/sludge cells was 14 nm while the LGOV-treated completely destroyed cells were 7 nm. Furthermore, the differences in surface area of untreated cells, LGO-, and LGOV-treated cells were 201.02% (Figure 4(a)), 1.58% (Figure 4(b)), and 3.48% (Figure 4(c)), respectively. As per SEM observation, the untreated bacterial cells were separated from each other which means that they had higher value/percentage of surface area per unit while LGO-treated cells appeared like the sludge so the difference in surface area per unit was much less (i.e., 1.58%). In LGOV-treated destroyed cells, the difference in surface area were more (i.e., 3.48%) than in the LGO-treated cells due to the presence of cellular debris. The three-dimensional structure of the *E. coli* cells also shows significant differences in the *Z* axis value which was 1000 nm/div (Figure 4(g)), 30 nm/div (Figure 4(h)) and 25 nm/div (Figure 4(i)) in untreated, LGO-treated and LGOV-treated samples, respectively. A reduction in cell height has also been recorded in the AFM observations of carvacrol [50] and PGLa-treated *E. coli* cells [43]. 

**Figure 5:**
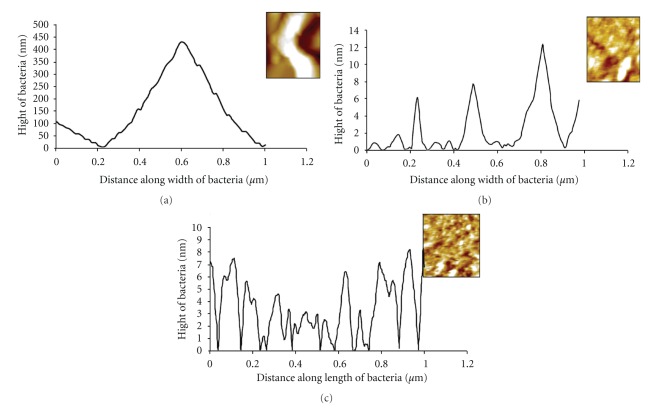
Graphical representation of atomic force microscopy results showing variation in height of *E. coli* cell along the width. (a) Untreated smooth cells (h 450 nm), (b) LGO-treated shrunken cells (h 14 nm). (c) LGOV-treated ruptured cells (h 7 nm).

Roughness analysis of differently treated *E. coli* cells has been observed with the atomic force microscope (AFM). The root mean square (rms) values of untreated, LGO-treated and LGOV-treated cells were 1.86 nm (Figure 4(a)), 2.33 nm (Figure 4(b)), and 3.32 nm (Figure 4(c)), respectively. This confirms that the effect of volatile compound (vapour phase) on the bacterial cell wall/membrane was more prominent than liquid phase/direct treated bacterial cells. Similar enhancement in the roughness of *E. coli* cells was observed by Da Silva and Teschke, [43] after treatment with PGLa. The roughness at the top surface of PGLa-treated bacterial cells increased substantially from 1 nm (for untreated bacterium) to 2.25 ± 0.03 nm. Furthermore, variation in height of the bacterial cell along the body length was observed in the present study (Figure 5) in the LGO- and LGOV-treated cells. By this graphical representation, we can estimate the higher effectivity of LGOV (Figure 5(c)) than LGO (Figure 5(b)) in terms of height versus surface roughness of differently treated cells. Similarly, La Storia et al. [50] noticed an increase in mean roughness in *E. coli* 32 cells from *≈*1 nm (for untreated bacterium) to 2.5 nm in carvacrol treated cells. The authors speculated that the action of carvacrol may render the components of the outer membrane (e.g., proteins and lipids) in Gram-negative bacteria more exposed to the external surface, causing an increase in roughness. In the present study, a more significant increase in roughness coupled with cell lysis and height reductions in LGOV-treated cells indicate that monoterpenes present in the LGOV cause higher damage in the gaseous phase. This could be attributed to better diffusibility and partitioning into membrane structures of bacteria in the gaseous phase [51].

Bacterial cell damage was observed using standard electron microscopy (TEM or SEM) in the past. Subsequently, some studies used AFM to study the antibacterial effect of synthetic antimicrobial compounds. Braga and Ricci, [52] used AFM for investigating the damage to bacterial morphology induced by an antimicrobial agent, cefodizime. Milder damages such as filamentation and bulge formation were observed in *E. coli *after exposure to sub-MICs of cefodizime while at supra-MIC levels bacterial flattening and emptying was observed. Supra-MICs of cefodizime induced the death of *E. coli* and led to lysis of the bacterium. Nevertheless, the damage was in terms of a hole on the surface, where the cell wall had disappeared to reveal the fine structure of the underlying cytoplasm, whereas the remaining part of the bacterium seemed to be intact. Furthermore, the height of the collapsed cell recorded by Braga and Ricci [52] was 160 nm. As compared to this study, the damage observed in the present work is much more extensive as complete rupturing of the cells is seen. The height of the treated cell is very significantly reduced (7–15 nm) as compared to the untreated cells (450 nm). Da Silva and Teschke [43] observed that the interaction of *E. coli* with PGLa initiated with the loss of surface stiffness and the formation of micelles while in the later stages only bacterial membrane residues (70 nm) could be observed. Recently, AFM was used to study the antibacterial effect of chitosan [39], aqueous garlic extract [53], eugenol [40], and carvacol [50]. The present study happens to be the first one to employ AFM for elucidating the effect of LGO and LGOV on *E. coli*.

## 4. Conclusion

The antibacterial effect of the LGO and LGOV against *E. coli* has been investigated employing different microscopic techniques. SEM, TEM, and AFM micrographs of the LGO- and LGOV-treated bacterial cells together show the evidence of rupture, cell lysis, membrane blebbing, and loss of cytoplasmic material.

Lack of details in SEM is overcome by TEM and AFM, with the latter providing vital information on the height of the cell and details of its topography. AFM and SEM examination revealed that rough surface morphology and shrinkage of the cell was apparent in the cells treated with LGO, when compared to the untreated ones. Loss of turgidity and leakage of the cytoplasm from the bacterial cells were also observed by TEM investigations. Loss of membrane integrity and damaged cell surface further supports the evidence that the mode of bactericidal action of LGOV against bacteria is through membrane disruption and further blocking of the cell growth. A further study under *in vitro *conditions is recommended to elaborate the antibacterial activities of LGO and LGOV for food preservation. 

## Figures and Tables

**Table 1:** Minimum inhibitory concentration (MIC) of *Cymbopogon* essential oils and their active components against *E. coli. *

S. no.	Antimicrobial compound/essential oil	*E. coli* strain	MIC	Reference
(1)	*Cymbopogon citratus*	—	0.12%	[30]

(2)	*Cymbopogon citratus*	LMG 8223	>0.8%	
(3)	*Cymbopogon martinii*	LMG 8223	0.2%	[31]
(4)	*Cymbopogon nardus*	LMG 8223	>0.8%	
(5)	*Cymbopogon winterianus*	LMG 8223	>0.8%	

(6)	Limonene	ATCC 25922	>20 mg/mL	
(7)	Linalool	ATCC 25922	1.25 mg/mL	[32]
(8)	*α*-pinene	ATCC 25922	2.0 mg/mL	
(9)	*β*-pinene	ATCC 25922	9.75 mg/mL	

(10)	Geraniol	ETEC 5041-1	20 *μ*g/mL	[33]
(11)	Geranyl acetate	ETEC 5041-1	0.5 mg/mL	

(12)	*β*-pinene	ATCC 13706	2.5 mg/mL	[34]
(13)	Caryophyllene	ATCC 13706	0.625 mg/mL	
